# Pharmacies: an important source of contraception for some adolescents, but not a panacea for all

**DOI:** 10.1080/26410397.2023.2221883

**Published:** 2023-07-07

**Authors:** Lianne Gonsalves, Asantesana Kamuyango, Venkatraman Chandra-Mouli

**Affiliations:** a Scientist, UNDP-UNFPA-UNICEF-WHO-World Bank Special Programme of Research, Development and Research Training in Human Reproduction (HRP), Department of Sexual and Reproductive Health and Research, World Health Organization, Geneva, Switzerland.; b Technical Officer, UNDP-UNFPA-UNICEF-WHO-World Bank Special Programme of Research, Development and Research Training in Human Reproduction (HRP), Department of Sexual and Reproductive Health and Research, World Health Organization, Geneva, Switzerland

**Keywords:** adolescents, contraception, pharmacy, youth, self-care

Meeting the sexual and reproductive health and rights (SRHR) needs of adolescents has risen substantially on the global agenda, accelerated by Sustainable Development Goal (SDG) indicators focusing on adolescent-specific birth rates and early marriage, as well as other SDG indicators for informed SRHR decision-making, HIV incidence, and satisfying contraception needs. On increasing adolescents’ ability to access contraception specifically, there has been important – albeit slow and uneven – progress in recent years.

The proportion of adolescent girls aged 15–19 years worldwide whose needs for family planning were satisfied by modern methods rose from 36% to 60% between 1995 and 2020.^1^ That said, wide variation can be seen across regions and within countries with regard to addressing unmet need for contraception. South Asia, sub-Saharan Africa, and the Middle East and North Africa have all seen steady increases in adolescent girls’ demand for contraception satisfied by modern methods over the past 25 years.^1^ However, fewer than 1 in 2 adolescent girls in these regions have their demand satisfied compared to around 3 in 4 girls in Latin America and the Caribbean, and Europe and Central Asia, and 88% of girls in North America.^1^

Even within the same country or community, adolescents are a heterogeneous group, with varying social, financial, legal, and economic circumstances that affect their willingness or ability to access contraception from a given outlet. As one example, social stigma around premarital sex means that unmarried adolescents’ ability to access subsidised or free contraceptives from government-run health services is very different from that of married ones. Many adolescents (irrespective of marital status) also express dissatisfaction with free or low-cost public health services, citing their lack of privacy, long waiting times, inconvenient opening hours, and erratic availability of the contraceptives they prefer (including, but not limited to, emergency contraception).^2^

Private pharmacies offer an alternative direct source of contraception for those discouraged by health facility services. Here, depending on the country, certain contraception methods have become available to adolescents either behind-the-counter (dispensing contingent on screening by a pharmacist) or over the counter (openly accessible). Pharmacies offering this access are appreciated by adolescents for their convenient locations, their privacy, speed, opening hours, and reliable stock.^3^ Many also appreciate that – particularly where over-the-counter access is available – they can get the contraceptives they want and leave promptly, without answering questions or being obliged to go through an education/counselling session.

Studies in several settings, including Ethiopia, Kenya, and Nepal, have shown that adolescents represent an important segment of the clientele for pharmacies and drug shops.^4–6^ However, this is not new. One of the authors of this commentary, who is now 64 years old, recalls vividly going to pharmacies in India far away from home (to avoid crossing paths with a family member or family friend) to try to buy condoms when he was a teenager. Shortly after emergency contraception first became available without a prescription in pharmacies in the United States, another author, now 34 years old, steered clear of the comprehensive services available at her university health centre, scraping together the money to pay for emergency contraception at a local pharmacy out-of-pocket.

Today, pharmacies and drug shops are receiving renewed attention. In countries around the world over the last 20 years, short-acting methods such as emergency contraception and daily contraceptive pills have become more broadly available in pharmacies without a prescription from a doctor.^7^ Additionally, innovation in contraceptive technologies, for example, through the development of subcutaneous depot medroxyprogesterone acetate (DMPA-SC) self-injectable contraception, has also helped broaden potential options for contraceptive users outside of a health facility. Finally, there has been growing enthusiasm within the SRHR community for self-care and task-sharing of contraceptive service provision to community distributors, pharmacy personnel, and users themselves.^8^ Attention to self-care interventions accelerated even more out of necessity in the height of the COVID-19 pandemic, when contraceptive services were paused, or deprioritised within many health systems.^9,10^

Studies also show, however, that while pharmacies are an important source of contraceptives for adolescents, they are not a panacea. First, pharmacies are limited in the range of contraceptive methods they can directly provide. There remain many settings where short-acting contraceptive methods cannot be dispensed to adolescents without a prescription from a doctor, making them unavailable directly through pharmacies (either over the counter or dispensed by pharmacists). One 2017/18 survey found that in only nine of 31 European countries were pharmacists able to provide emergency contraception to adolescents.^11^ Also, for contraceptive users of all ages, long-acting methods remain the domain of health facilities.

Second, pharmacies are limited in their ability to consistently provide information and counselling services. One study using mystery shoppers to test actual availability of emergency contraception to adolescents in France found that – contrary to regulations – fewer than half of pharmacies who dispensed provided information on the drug’s use, and none provided additional counselling.^12^ Quality of SRHR services dispensed in pharmacies can be variable for many reasons, including environmental strains (busyness, lack of space or privacy), as well as pharmacist or client discomfort.^3^ A shy purchase of condoms in a pharmacy, therefore, could be deeply appreciated for its brevity but provide no opportunity to sensitise adolescents to the full range of contraceptives available.

Third, while private pharmacies can (with deregulation) increase accessibility of many short-acting methods relied upon by adolescents, access is not free, and purchases (particularly without a prescription) are likely to be paid for out-of-pocket. A Canadian Paediatric Society statement advocating for no-cost contraception for young people in Canada warned that existing available public and private health plans had variable coverage of contraceptive methods. Even if an adolescent was covered by a private plan, accessing contraception through that coverage would trigger an automatic report to the policy holder (likely a parent), meaning that in practice adolescents would need to pay out-of-pocket to ensure confidentiality.^13^ There is convincing evidence that for adolescents, who may not have independent sources of income, cost can be a prohibitive barrier. A recent study by Kamuyango and others showed that young women aged 15–24 from urban areas in low-income countries were more likely to access private sources of contraception. On the other hand, those from poor households were less likely to obtain contraceptives from private pharmacies and clinics than their more privileged counterparts in sub-Saharan African countries.^14^ These findings are consistent with the high levels of both unmet need for contraception, and of unsafe abortion among adolescents who live in rural areas, as well as those from the poorest households.

What does this mean for pharmacies as contraceptive resources for adolescents? In their current form, pharmacies are valuable contraceptive sources for many adolescents, with their service qualities making them organically “adolescent-friendly”. However, pharmacy services should not be mistaken for a “silver bullet” intervention that will address the continuing unmet need for contraception among this age group.

Instead, a “whole of market” approach, with outreach to and engagement with the full spectrum of available contraceptive outlets across public, private, and not-for-profit sectors, can help governments develop a supportive net of contraceptive services for their heterogeneous adolescent population.^15^ Engaging with adolescents themselves through well-designed (and ethically sound) studies or civil society consultative mechanisms, can provide opportunities to understand the diverse and evolving needs of different groups of adolescents. This, in turn, can inform the (re)design of responsive services, capitalising on the different strengths of various contraceptive delivery outlets. Government-run services, for example, could be more varied in delivery. Drawing from the appealing qualities of pharmacies, public facilities might have an option for free, “grab-and-go” short-acting contraception in one private area of a clinic, in addition to the existing options for more extensive contraceptive counselling and service delivery. Additionally, governments could choose to support adolescents’ abilities to access contraception at pharmacies by eliminating or reducing financial barriers to access. France made international headlines recently on this front, when it announced that, as of 2023, emergency contraception would be made available for free at French pharmacies for all women aged 25 and under.^16^ The Taranaki region in New Zealand also recently announced plans to provide free emergency contraception through pharmacies for young people aged 12–24.^17^ Alternatively, governments could support pharmacies in providing services free of charge to those adolescents who cannot afford to pay, and charging an affordable fee for those who can.

Finally, expanding adolescents’ and young people’s access to contraception remains tightly intertwined with broader conversations on the “right to health” including SRHR, and universal health coverage. However, the appeal of pharmacies as contraceptive access points for adolescents in both high- and low- and middle-income countries, should also alert us to continued social inequalities related to gender and our continuing global discomfort with S-E-X. This conversation around pharmacy access, therefore, turns a mirror on the shortcomings of governments, the health system, and SRHR field, on communities, and on the adults who love and want the best for the adolescents and young people in their lives. If all or some groups of adolescents continue to lean heavily into out-of-pocket purchases of contraception, particularly if free or “adolescent-friendly” services are available nearby, it can serve as a reminder that we in the SRHR community can do more to create environments that truly empower young people to make decisions about their sex lives that promote safety, consent, and satisfaction. The onus for bringing about improvements in their sexual and reproductive health cannot rest solely with adolescents themselves.
